# Dealing with Nap-Core Sandwich Composites: How to Predict the Effect of Symmetry

**DOI:** 10.3390/ma12060874

**Published:** 2019-03-15

**Authors:** Giap X. Ha, Manfred W. Zehn, Dragan Marinkovic, Cristiano Fragassa

**Affiliations:** 1 Department of Structural Mechanics, Institute of Mechanics, Technical University of Berlin, 10623 Berlin, Germany; xuan.g.ha@campus.tu-berlin.de (G.X.H.); manfred.zehn@tu-berlin.de (M.W.Z.); dragan.marinkovic@tu-berlin.de (D.M.); 2 Department of Industrial Engineering, University of Bologna, 40136 Bologna, Italy

**Keywords:** textiles, material layers, composite properties, symmetry, numerical models

## Abstract

The behavior of nap-core sandwiches was investigated with a special focus on the effect of symmetry in nap cores. A nap-core is, in general terms, a 3D-formed hollow structure made of knitted textile impregnated by a thermosetting resin. The molding process determines if the nap-core is double-sided (symmetric) or single-sided. The sandwich with nap-core owns various remarkable properties of a novel lightweight material, but the nap-core’s complex structure makes the prediction of these properties a difficult task. While the analysis of a single-sided nap-core sandwich has been presented by the authors before, this study is focused on the simulation of symmetric nap-core sandwich. Overall, performance of the structure is examined with respect to several loading conditions. The simulation approach invokes a typical homogenization scheme to find the engineering properties of the nap-core’s fabric with least computational time and memory resources. Results from experiments and simulations exhibit a good compatibility, which prove the fitness of the modeling method.

## 1. Introduction

It did not take much time for composite materials to conquer the field of lightweight structures engineering. Their exquisite material properties together with the additional, unique ability of tailoring those properties have deservedly brought this reputation to them [1]. Whatever properties are required from a specific structure, with proper engineering on the material level, composite materials can provide an adequate response to a vast range of challenges and needs [2]. The scope of their application is rather wide covering automotive and aeronautical industries, sporting goods, biomedical materials, and so on. A huge amount of research is already able to deal with aspects related to the design of solutions for parts made of composites [3,4] including the development of methods and tools for their material modeling [5,6], or coupled-field effects in cases when composites involve multi-functional materials [7,8], criteria for their failure [9,10], to name but a few areas of work.

Besides, the application of textile materials in composites is extremely common because of their convenience, especially related to their very high strength compared to the weight. Mostly, they appear like reinforcement in membranes and composites, or even as essential parts in dust filters [11,12]. In recent time, their application has been extended to lining and interior covers of vehicles in the form of sandwich-structured composites, a new family of composite materials also identified as core materials, consisting of a thick but lightweight and hollow core placed between two thin outer layers that are rather stiff (i.e., face sheets or skins) [13]. 

Given the exquisite relation between performance and weight, combined with the general usability of these materials, the sandwich type of composites is employed more and more in relevant applications such as automobiles, aircrafts, and spaceships. 

By changing the parameters of sandwich composites, their general mechanical behavior can be adapted to cover the need of various applications [14]. 

Lining elements have been produced in the past, mostly using nonmetal sandwich materials where a core is a honeycomb or a foam covered and protected by outer layers, usually made of laminated fabric composites [15]. However, the introduction of nap-core has provided an additional great choice for core materials. The development of the nap-core sandwich composite has been started in the 1980s and continued by Brandenburg Technical University and Fraunhofer Institute and InnoMat GmbH in Germany up to now because of its noteworthy structural merits [16].

**Figure 1:**
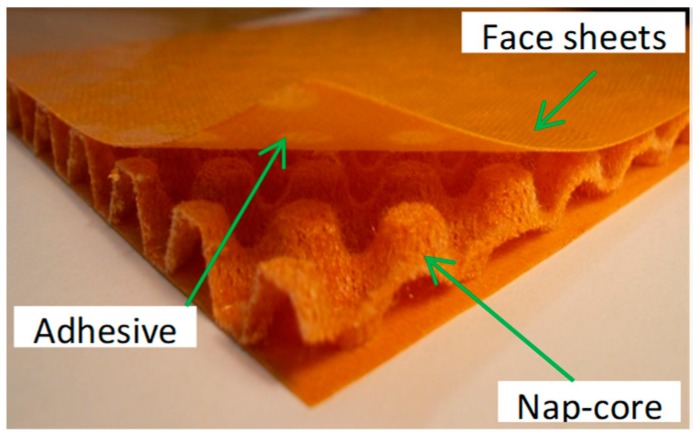
Layers and constituent parts of a symmetric nap-core sandwich.

Basically, a nap-core is made of a two-dimensional knitted fabric impregnated by thermosetting resin, which further undergoes deep-drawing and curing processes with the aim to assume a steady 3D shape (characterized by a height between 5 and 10 mm) as a mixture of periodic-distributed identical naps. Knitted fabric is selected because of its distinct drapability that allows the cores to conform to sharply curved surfaces without any crimp. The nap-core has a fibrous pattern different than the one of a continuous laminate where the resin has a protecting role as coating layer. It is the resin complementing mechanical sturdiness and durability to natural looseness of the knitted fabric. To form the sandwich, one nap-core is firmly bonded with two thin stiff fiberglass-laminated skins at the top and the bottom [17]. The skins improve the bending stiffness of the nap-core. Essentially, the final product consists of a nap-core sandwich structure, a layered material where every part is also made of a fiber-resin reinforced composite (see Figure 1). 

Nap-core sandwich composites possess many distinguished properties from the mechanical, physical, and chemical aspects that are detailed in References [18,19]. The most noticeable ones are:

- No transition or separation between fibers or yarns is evident in the material.
- Even if the cured fabric is discontinuous, it behaves as a thin and flexible cloth sheet.
- The deformation of the nap-core is permanent, able to offer a specific shape,
- With specific strengths lower than honeycombs or foams with similar density, nap-core sandwiches are also cheaper in production.
- There are voids among naps and on the fabric of the nap-core, so its sandwich provides drainage or ventilation for fluids. Also, the integration of ducts and wires can be easily made.
- The flexibility of nap-core sandwiches allows them to become easily curved in applications.
- Changes in the materials and geometries of the nap-core permit the creation of different sorts of sandwiches, adjusting mechanical properties in the way to fit each specific requirement.

**Figure 2:**
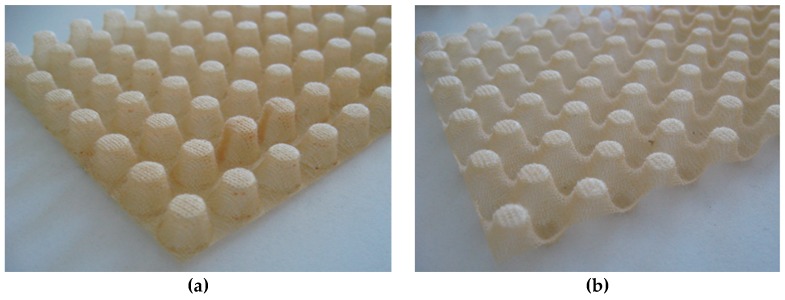
Single-sided nap-core (**a**), and symmetric nap-core (**b**).

Two major types of nap-cores can be distinguished based on their layout: single-sided and double-sided (symmetric) nap-cores (see Figure 2). While the former is more suitable for handcraft, the latter is more convenient for automatic production [20]. The former is shaped between a positive tool and a negative tool, while the latter is shaped between two positive tools. Not only can the symmetric nap-core facilitate an automated production process much better, it is also more advantageous at complex drapability since the naps can converge and diverge on both sides. However, the geometry of the symmetric nap-core may limit it to usage of pins considerably smaller than those used for a single-sided nap-core, plus its deep-drawing molds are more expensive [21]. Especially, symmetric nap-core sandwich offers appreciable mechanical properties owing to its shape. The diameter of the naps’ top side is rather small compared to single-sided nap-core types. Hence, the density of naps inside symmetric nap-core is very high, thus providing higher resistance to the sandwich when the knitted fabrics have the same materials and thickness. 

As they are in the early phase of development, the current typical application of nap-core sandwich is for the roof, wall, and hulk of vehicles in aerospace and automotive industries. These components are not designed to endure large loads or strong impacts. However, with all the precious properties mentioned above, it is expected that nap-core sandwiched structures will exhibit much better strength and resistance in the future. In this sense, numerical modeling methods of the nap-core sandwich are indispensable in the structural analysis of parts made of them. This requires a thorough consideration of the anisotropic material and non-periodic mesoscopic structure of the nap-core as well as residual stress in its knitted fabric.

The unique properties and internal structure of a nap-core sandwich make it rather challenging for both testing and modeling. The authors found that there is rather limited literature that considers this important new material. Most of the literature is related to testing. Bernaschek [22] tested mechanical properties of nap-cores and honeycombs and compared them. Gerber [19] tested mechanical properties of single-sided and symmetrical nap-cores and compared them from the perspective of their use in lightweight applications. In a further work, Gerber et al. [13] studied the behavior of honeycomb and symmetrical nap-core sandwich structures exposed to an impact load. On the modeling side, Ha and Zehn [17] and Ha et al. [18] gave a detailed discussion of the problems related to building appropriate Finite Element models of nap-core sandwich composites and simulation of their mechanical behavior. They also suggested some feasible approaches to simulate the two types of this structural material. The simulation results showed good agreement with the experimental ones. All types of the considered sandwich samples in those analyses were single-sided nap-cores. The present work expands the use and confirm the validity of the applied approach by applying it to simulate sandwich composites fabricated as symmetric nap-cores. 

## 2. Materials and Methods 

In this paper, a typical symmetric nap-core type will be modelled and investigated. It is named P2-8, in which P stands for phenol formaldehyde resin; “2” means double-sided; and “8” means the height of the nap-core is 8 cm. P2-8 nap-core has a fabric thickness of 0.45 mm, a volume density of 41 kg/m^3^, and it consists of 50% fiber (80% aramid, 20% polyester) and 50% phenol formaldehyde resin. These proportions of the constituent materials are necessary in calculating the effective mechanical behaviour of the yarns of the nap-core’s fabrics for the purpose of simulation. Besides, the mass density of the P2-8 nap-core sandwich, including the nap-core and two face sheets, is around 175 kg/m^3^. The material was provided by the Institute of Mechanics of Technical University of Berlin (Berlin, Germany), Fraunhofer pyco (Teltow, Germany), and InnoMat GmbH (Teltow, Germany).

**Figure 3:**
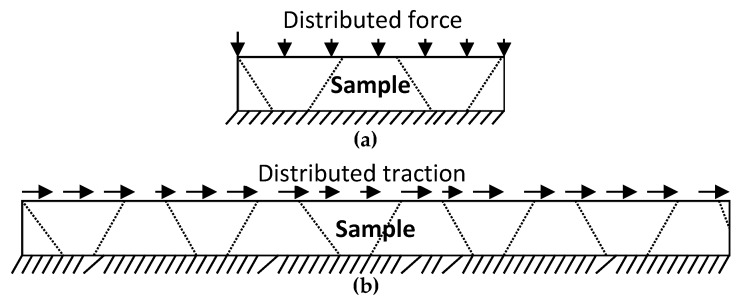
Compression test (**a**), and shear test (**b**).

**Figure 4:**
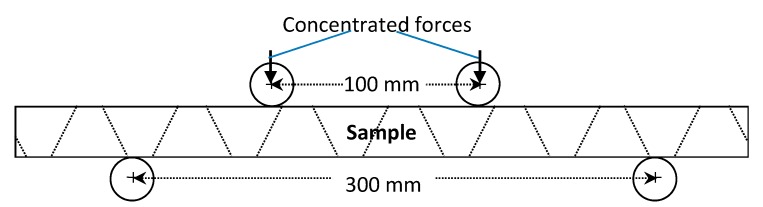
Four-point bending test.

Three different tests of sandwich panels involving the P2-8 nap-core were done: compression, shear, and four-point bending. Beside these tests, which aim at an investigation of the mechanical deformational behavior, a drum peel test was also conducted but it was only for the determination of the adhesive parameters. The experimental standards and dimensions for the sandwich samples are given in Table 1, while Figure 3 and Figure 4 depict the boundary conditions of the conducted experiments. The total thickness of samples is 8 mm. 

## 3. Simulation Approach

### 3.1. Modelling the Complexity.

Because the nap-core sandwich structure is very complicated, rather detailed modeling would be very cumbersome and prohibitively expensive. Hence, the authors opt for an approach in which the face sheets are modeled as shells. Having in mind a rather small thickness compared to the span, the transverse shear effect can be neglected [21] and the nap-core can also be modeled as a thin shell imposing the need for the in-plane material properties only. Hence, the number of necessary engineering parameters is reduced from nine to four and, generally speaking, they can be determined via experiments. Namely, standard DIN EN ISO 13934-1 is typically applied to measure two tensile moduli and one Poisson’s ratio, while a picture frame shear test is used to determine the corresponding shear modulus. 

The cohesive layers are modeled as thin bonding layers [18]. Since no data are available able to characterize the nap-core sandwich’s adhesive material, its main parameters have been evaluated by authors via peeling tests on the material samples. These values, reported in Table 2, are in line with several classic studies on cohesion such as those by Alfano [23], Davila [24], or Linde [25]. 

Then, in accordance with these studies, and as expressed in Reference [18], the following assumptions are introduced for the cohesive elements:

- In the numerical model, the cohesive elements are implemented using the “traction-separation” type of cohesive section.
- The damage initiation can be considered as following the quadratic traction-interaction failure criterion (quads damage).
- The damage evolution is described using an energy-based law, while damage propagation can be found by using the power law criterion.

The specific material data of the nap-core sandwiches’ adhesive are presented in Table 2, where:

E_nn_—Young’s modulus along the normal direction
G_ss_—Shear modulus along the local first direction
G_tt_—Shear modulus along the local second direction
N_0_—Nominal stress in normal-only-mode
T_0_—Nominal stress in the first direction
S_0_—Nominal stress in the second direction of the cohesion damage
G_1C_—Fracture energy in normal mode
G_2C_—Fracture energy for the first direction in shear mode
G_3C_—Fracture energy for the second direction in shear mode
α—Power of the damage evolution, equal to 1.45 for all the nap-core sandwiches (note that the unit of fracture energy is J/m^2^ which is equivalent to N/m).

The elasticity values (E/E_nn_, G_1_/E_ss_, and G_2_/E_tt_) can be chosen quite freely as long as the model converges. However, the values of quads damage (N_0_, T_0_, and S_0_) and damage evolution (G_1C_, G_2C_, and G_3C_) are calibrated gradually since they determine the start point and intensity of the damage. Namely, if the values of quads damage increases, the damage will start at a point having higher force and higher displacement. If the values of damage evolution increases, the damage will develop more slowly.

**Figure 5:**
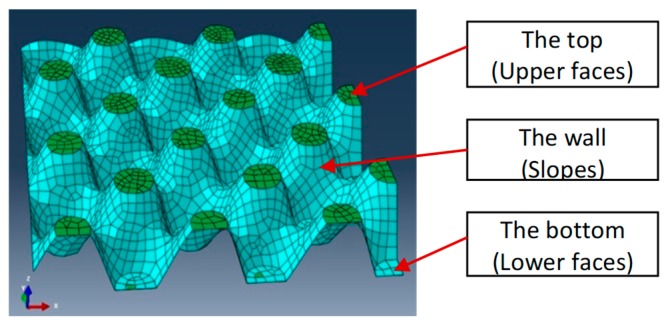
Top, bottom, and wall faces of the symmetric nap-core.

The nap-core is simulated as a homogeneous 3D shell presenting the geometry of the cured and stamped knitted fabric. Hence, on the macroscopic level, the geometry of the nap-core remained the same, while at a mesoscopic level it was modified from the non-continuous knitted fabric to a continuous shell (see Figure 5). In doing that, it is important to keep its effective mechanical properties at the macroscopic scale, i.e., to determine four engineering constants in this case. To find their values, a homogenization scheme is invoked to homogenize the knitted fabric of the nap-core’s wall. 

Several methods have been proposed to determine the homogenized properties: rules of mixtures [26], self-consistent scheme (SCS) [27], generalized self-consistent scheme (GSCS) [28], asymptotic homogenization (AH) [29], and Representative Volume Element (RVE) [30]. The last one is chosen by the authors due to its simplicity and effectiveness, which can readily take advantage of the computing power of numerical software such as Abaqus, Version 6.14.2. [31]. 

The homogenized material properties are then obtained using a computational model of a unit cell of the real nap-core’s knitted fabric. Basically, the RVE method picks a carefully chosen RVE from the observed periodic structure, applies the periodic boundary conditions, and determines the effective mechanical properties based on the equivalence of the strain energy with that determined for the same sample only considered to be made of homogenous material [32].

As analyzed in a previous publication [17], the results indicate that the material properties of the nap-core’s wall are the most influential, as they determine approximately 90% of the resulting force and displacements in the tests. Therefore, the material parameters of the upper (top) and the lower (bottom) nap-core faces can assume the values of the wall without a considerable effect on the results. Like the face sheets, the geometry of the nap-core’s wall allows to consider it as a thin-shell structure, so again only four of nine engineering constants are needed: the elastic moduli, E_1_ and E_2_, the shear modulus, G_12_, and the Poisson’s ratio, ν_12_, for the in-plane directions.

The mesh density of the Finite Element (FE) models is determined through a convergence analysis. The biquadratic quadrilateral shell element S8R from the Abaqus element library was used. Rounded to thousands, the FE meshes yielded converged results where 15,000 elements (46,000 nodes) were used for the compression test, 30,000 elements (93,000 nodes) for the shear test, and 61,000 elements (187,000 nodes) for the bending test. 

### 3.2. Homogenization Scheme

The process of homogenizing the nap-core’s fabric is presented as follows [17].

#### 3.2.1. Choice of RVE for the Nap-Core’s Knitted Fabric

Even if there are several approaches to make a proper selection of RVE, in practical terms, an ideal RVE would be the smallest possible one, with simple geometry, chosen so as to keep the all the material parameters of the nap-core wall irresponsive to boundary conditions. Because the nap-core’s wall is the dominant portion, its RVE can represent the whole nap-core. Figure 7a shows an RVE taken from the nap-core’s wall.

#### 3.2.2. Setting the FE Model of RVE 

In this step, an FE model was prepared for the chosen RVE and the mechanical properties were assigned to constituent materials. To create an RVE, it is necessary to determine the geometry of the warp yarn and the weft yarn using a minimum of nine discrete points for each yarn. These points are used for reconstructing 3D splines in Abaqus as a way of representing the swept directions of fibers. A microscope is used for this purpose. The yarns should be created as swept solids or beams with the transverse shear stiffness to give accurate results. The final precision of the homogenization procedure significantly depends on the mesh density. Therefore, the size of finite elements used to discretize the RVE was gradually decreased until the converged result was obtained. Finally, the fibrous RVE was embedded into a cubic box of a soft elastic material, which was crucial for the imposition of a boundary condition on the RVE.

#### 3.2.3. Boundary Conditions and Computation

Within the framework of the RVE homogenization approach, three different sets of boundary conditions can be used: (i) linear displacement boundary condition (Dirichlet condition), (ii) constant traction boundary condition (Neumann condition), and (iii) periodic boundary condition. 

Among them, the latest represents the favored choice in numerous numerical studies as the one that offers a rather good convergence rate of solutions. This refers to periodic structures, even in the absence of geometric periodicity on the micro level [32,33]. 

**Figure 6:**
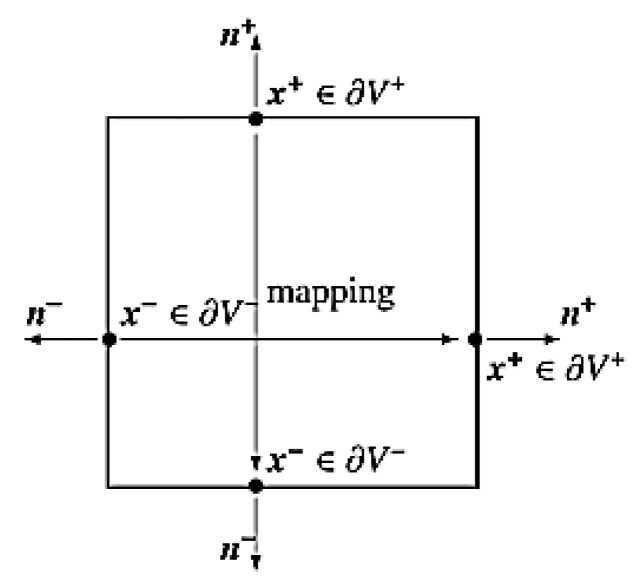
Periodic boundary conditions.

**Figure 7:**
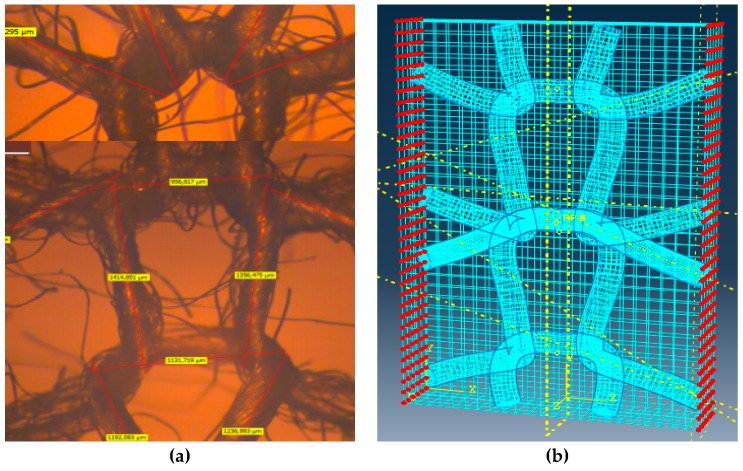
The real RVE (**a**), and the simulation RVE with periodic boundary conditions (**b**).

A periodic boundary condition requires identical FE discretization on each pair of opposite RVE surfaces (see Figure 6). All couples of opposite nodes are supposed to have the same displacements that can be described as

u_i_^k+^ = ε_ij_^0^ x_j_^k+^ + u_i_^*^, (1)

u_i_^k−^ = ε_ij_^0^ x_j_^k−^ + u_i_^*^, (2)

u_i_^k+^ − u_i_^k−^ = ε_ij_^0^ (x_j_^k+^ − x_j_^k−^) = ε_ij_^0^ Δx_j_^k^ (3)

where indices k^+^ and k^−^ denote the k^th^ pair of corresponding nodes on two opposite boundary surfaces of the RVE, and Δx_j_^k^ is the distance between the two nodes in the initial configuration. Because Δx_j_^k^ is constant for each pair of the parallel boundary surfaces, the right side of Equation (3) becomes constant with a specified ε_ij_^0^. Such equations can be applied to FE analysis as equation constraints [34,35]. Because the number of node pairs is large, a Python script was coded to identify the pairs and apply the equation constraints automatically. The RVE with the node sets for the periodic boundary condition is shown in Figure 7b.

Before defining constraints and performing calculations, the mechanical properties of the “nap-core’s fiber/resin bundles” are needed. Each bundle makes a yarn consisting of a fiber surrounded by resin. Their mechanical properties can be determined by applying the rule of mixtures [36]:

(4) $$\{\begin{array}{c} E_{L}=E_{f}V_{f}+E_{m}\left(1-V_{f}\right)\\ \frac{1}{E_{T}}=\frac{V_{f}}{E_{f}}+\frac{\left(1-V_{f}\right)}{E_{m}}\\ \nu_{\mathrm{LT}}=\nu_{f}V_{f}+\nu_{m}\left(1-V_{f}\right)\\ \frac{1}{G_{\mathrm{LT}}}=\frac{V_{f}}{G_{f}}+\frac{\left(1-V_{f}\right)}{G_{m}} \end{array}$$

where:

E_f_ and E_m_ are the Young’s moduli of the fiber and the matrix
E_L_ and E_T_ are the effective longitudinal/transverse elastic moduli
V_f_ is volume fraction of fiber in the bundle.

Although the result is of acceptable accuracy for the longitudinal values (E_L_, G_LT_, and ν_LT_), it is problematic for the transverse value (E_T_). In 2000, Jacquet et al. suggested a more appropriate formula for E_T_, which was verified to yield a very realistic result [26]. They extracted E_T_ based on improved modeling of the structure as:

(5) $$E_{T}=\frac{E_{f}E_{m}}{E_{m}+E_{f}\left(1-\sqrt{V_{f}}\right)/\sqrt{V_{f}}}+\left(1-\sqrt{V_{f}}\right)E_{m}$$

The parameters of the nap-cores’ constituent materials (fibers and matrices) and the resin content ratios are introduced in Table 3. The elliptic sections of the yarns are shown in Table 4.

Finally, based on the result of numerical analysis using RVE, the following homogenized engineering constants of P2-8 symmetric nap-core were obtained: E_1_ = 43.85 × 10^7^ (Pa), E_2_ = 34.26 × 10^7^ (Pa), G_12_ = 121.15 × 10^7^(Pa), and ν_12_ = 0.28. 

## 4. Results and Discussions

This section presents the experimental and simulation results obtained with samples made of P2-8 nap-core sandwich with the geometry as presented in Section 2. 

### 4.1. Compression

**Figure 8:**
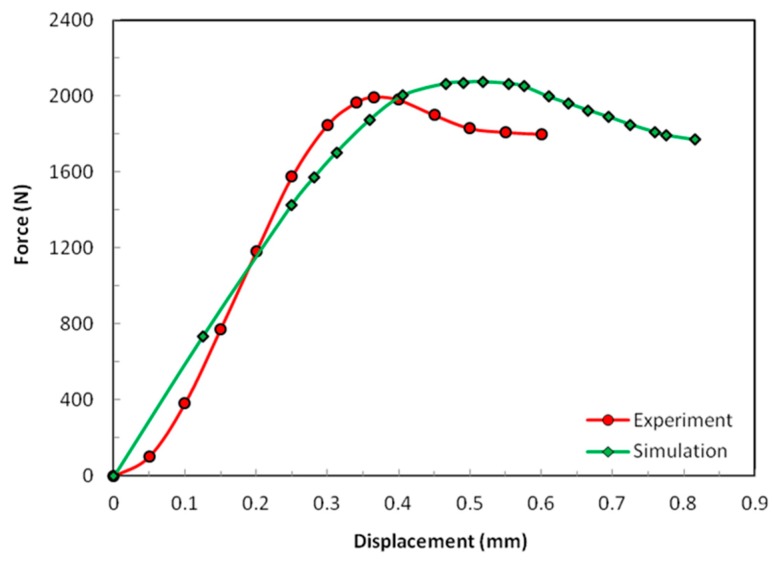
Compression results of the test and the simulation.

From the experimental results shown in Figure 8, one can recognize a few characteristics in the mechanical behavior of the sandwich sample involving P2-8 nap-core. There was a short establishment period until the displacement reached the value of approximately 0.075 mm and the force of approximately 100 N. Afterwards, the sample deformed almost linearly until a force of approximately 1600 N. The sample buckled when the displacement reached the value of 0.36 mm and the maximum force was nearly 2000 N. Interestingly, the force remained relatively high after the buckling, thus providing further solid resistance even after buckling. 

In Figure 8, it is evident how the simulation predicts the buckling force with a satisfying accuracy, while the estimated stiffness (homogenized moduli) is, though obviously smaller, still acceptable keeping in mind that it was dominated in this case by the nap-core side walls, which buckle, while the skin layers and the nap-core top and bottom faces deformed only negligibly in this case. As already seen, the nap-core stiffness was also a result of simulation, and not of the experiment, as it was obtained based on the RVE taken from one location on the nap-core wall.

The difference in the so-obtained and actual stiffness was responsible for the discrepancy between the numerical and experimental values of the displacements at buckling. The establishment period of the experiment was not reflected by the simulation since it was a very complicated effect that characterized the initial deformation of the nap-core until the whole surface of the sample started to resist the external load.

### 4.2. Shear

**Figure 9:**
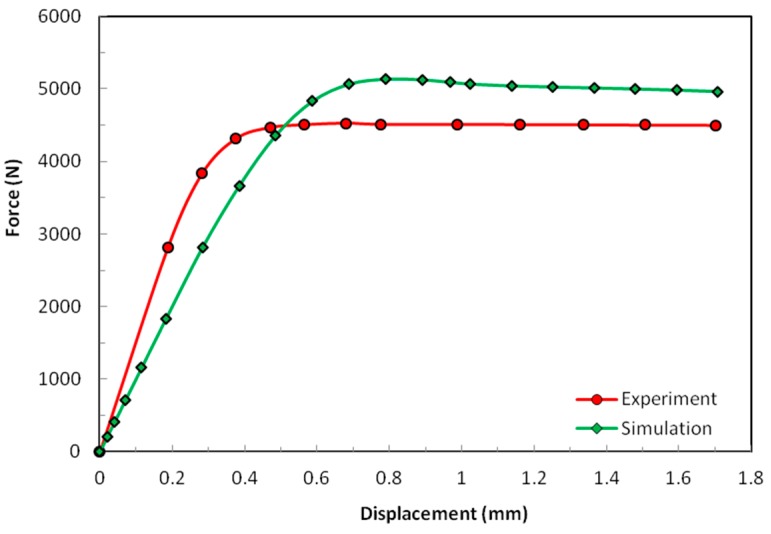
Shear results of the test and the simulation.

In Figure 9, the experimental sample does not show a clear establishment period. It deformed linearly from the start until the force of approximately 3500 N. After that, the sample started exhibiting nonlinear behavior. The maximum force was reached for displacement between 0.6 mm and 0.7 mm. In this case, the difference in the numerically estimated and actual stiffness was reflected in the difference between the maximum shear force obtained experimentally and numerically. On the other hand, with careful observation, one would notice that the displacements at the maximum shear force were relatively close. This difference in the way how experimental and simulation results in shear and compression compared to each other is to be attributed to the different buckling forms of the nap-core in those two cases. Additionally, in compression, the buckling changed the nap geometry in a way that resulted in a not large but still noticeable drop of force. This was not the case in shear case and the force remained almost constant.

**Figure 10:**
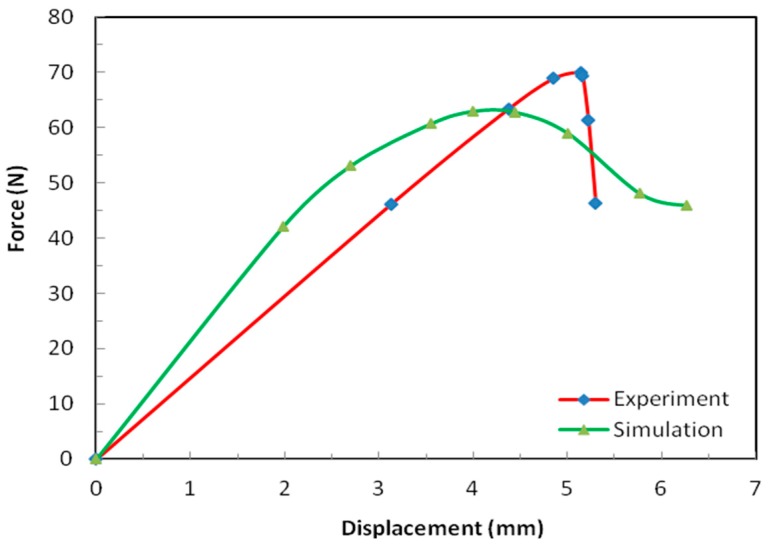
Four-point bending results of the test and the simulation.

Figure 10 shows the results obtained in the case of four-point bending test. This configuration was the preferred one considering that the alternative three-point bending test would result in the maximum bending moment over a rather limited domain of the sample. With respect to the size of that domain, the internal structure of the sample (nap-core) was inhomogeneous, and hence, the obtained result would strongly depend on the location at which the maximum bending moment occurred in the sample. For four-point bending, a larger area (which could be arbitrarily chosen) was exposed to the maximum bending moment. 

In the test, the deformation proceeded in an almost linear manner until the point of the maximum force. After that point, the sample failed as sudden local debonding occurred between the nap-core and skin layers. In contrast to the previous two tests, the numerical model demonstrated in this case higher stiffness compared to the stiffness observed in the experiment. In the first two test cases, the nap-core top and bottom faces together with the skin layers played a minimal role, i.e., the structural response was determined practically entirely by the nap-core walls (Figure 5). 

However, in bending, the nap-core top and bottom faces, as well as the skin layers, as outer structural layers, had a large influence, and it is reasonable to assume that this was the reason for the mentioned difference. 

The results for the forces obtained by the experiments and simulations in the three conducted tests are summarized in Table 5. 

The difference in the experimental and simulation results was under 15% for all conducted tests. Though in engineering terms the difference is not very small, besides the fact that this study represents one of the first attempts to model such a material, one also has to keep in mind that:

(1) The material structure is of extreme complexity.
(2) Simplifications were introduced in the models: complex, inhomogeneous composites were modeled as shells with homogenized mechanical properties, whereby the homogenization was done by means of simulation with the material properties of constituents taken from literature.
(3) Changes in the nap-core’s inner structure at higher deformations and material nonlinearities were neglected in the models.

In general, despite those differences in the results, the simulation based on the developed models reflects the actual mechanical behavior of the samples acceptably well. This means that they can be used as a basis for further numerical investigations of nap-core sandwich composites as means for virtual testing of different scenarios and thus to improve their mechanical properties. 

## 5. Conclusions

The results obtained in the conducted experimental tests and numerical simulation allow us to draw several noteworthy conclusions.

The approach to FE modeling and simulation of single-sided nap-core sandwiches based on the RVE homogenization [17,18] can also be applied for symmetric nap-core sandwiched structures. The homogenization allows modeling of complex resin-impregnated knitted fabric as a thin shell, thus reducing the numerical effort dramatically and saving a great deal of time and costs. In contrast, full FE modeling of the nap-core would be prohibitively expensive because of its complicated geometry though would demand a very fine mesh of solid elements, demanding extremely large storage capacity and computational time. The simulation model reflects the experimental sample behavior with acceptable quality, so the mechanical behavior of the symmetric nap-core sandwich under load cases can be predicted with acceptable accuracy.

The implementation of the RVE homogenization method is relatively simple and inexpensive, thus representing a powerful tool to investigate the influence of constituents (choice of material, their content, etc.) of the nap-core sandwich to its global behavior. For example, if yarn sections or the materials of the fiber and resin change, the effective mechanical and strength properties of the material will be modified as well. As a result, the nap-core sandwich performance and usability will be altered.

In the future work related to modeling, fine tuning of the material parameters, which could be done by determining the homogenized/effective material properties in tests and model identification techniques, should be done to improve the accuracy of results. This would allow further parametric investigations based on simulation models in order to optimize the design of this composite. This would certainly broaden their field of applications.

## Figures and Tables

**Table 1:** Experimental data for P2-8 nap-core sandwich mechanical tests.

Test	Standard	Sample Size Length (mm) × Width (mm)	Test Speed (mm/min)
Compression	D 3410/D 3410M–03	50 × 50	10
Shear	DIN 53 294	200 × 50	1
Four-point bending	DIN 53 293	400 × 50	10

**Table 2:** Cohesion parameters of sandwiches with nap-core type P2-8 (adhesive thickness = 0.01 mm).

E_nn_ = 1 × 10^6^ (Pa)	G_ss_ = 1 × 10^6^ (Pa)	G_tt_ = 1 × 10^6^ (Pa)
N_0_ = 5 × 10^6^ (Pa)	T_0_ = 4 × 10^6^ (Pa)	S_0_ = 4 × 10^6^ (Pa)
G_1C_ = 300 (N/m)	G_2C_ = 400 (N/m)	G_3C_ = 400 (N/m)

**Table 3:** Mechanical properties of the constituent materials.

Constituent	Young’s Modulus (GPa)	Poisson’s Ratio
Aramid	70	0.25
Polyester thermoset	56.8	0.33
Phenol Formaldehyde	2.9	0.42

**Table 4:** Average values (in mm) of the major and minor axis of the yarn sections (P2-8 nap-core).

Warp Yarns		Weft Yarns	
Major Axis	Minor Axis	Major Axis	Minor Axis
0.18	0.125	0.20	0.14

**Table 5:** Simulation vs experiment in terms of maximum forces.

Sample Size (mm)	5 × 5	20 × 5	50 × 5
Max Force (N)	Compression	Shear	Bending
Experiment	1995	4528	70.1
Simulation	2245	5123	60.7
*Difference*	12.5%	13.1%	−13.36%
